# Approaches to Identify the Value of Seminatural Habitats for Conservation Biological Control

**DOI:** 10.3390/insects11030195

**Published:** 2020-03-20

**Authors:** John M. Holland, Philippe Jeanneret, Anna-Camilla Moonen, Wopke van der Werf, Walter A.H. Rossing, Daniele Antichi, Martin H. Entling, Brice Giffard, Herman Helsen, Mark Szalai, Carlo Rega, Caroline Gibert, Eve Veromann

**Affiliations:** 1 Farmland Ecology Unit, Game and Wildlife Conservation Trust, Fordingbridge SP6 1EF, UK; 2 Agroecology and Environment, Agroscope, CH-8046 Zurich, Switzerland; philippe.jeanneret@agroscope.admin.ch; 3 Scuola Superiore Sant’Anna, Agroecology Group, Institute of Life Sciences, Via Santa Cecilia 3, 56127 Pisa, Italy; moonen@sssup.it; 4 Wageningen University & Research, Crop Systems Analysis, Droevendaalsesteeg 1, 6708PB Wageningen, The Netherlands; wopke.vanderwerf@wur.nl; 5 Wageningen University & Research, Farming Systems Ecology, Droevendaalsesteeg 1, 6708PB Wageningen, The Netherlands; walter.rossing@wur.nl; 6 Centre for Agri-environmental Research “Enrico Avanzi”, University of Pisa, Via Vecchia di Marina 6, San Piero a Grado 56122, Pisa, Italy; daniele.antichi@unipi.it; 7 iES Landau, Institute for Environmental Sciences, University of Koblenz-Landau, Fortstr. 7, D-76829 Landau, Germany; entling@uni-landau.de; 8 Bordeaux Sciences Agro, INRAE, UMR 1065 Santé et Agroécologie du Vignoble, University of Bordeaux, F-33170 Bordeaux, France; brice.giffard@gmail.com; 9 Plant Protection Institute, Szent Istvan University, Pater K. str. 1, Szent Istvan University, H-2100 Gödöllő, Hungary; Herman.Helsen@wur.nl; 10 Wageningen University & Research, Wageningen Plant Research, Lingewal 1, 6668LA Randwijk, The Netherlands; Szalai.Mark@mkk.szie.hu; 11 European Commission, Joint Research Centre (JRC), Via E. Fermi 2749, Ispra, VA, Italy; carlo.rega@ec.europa.eu; 12 SOLAGRO, 75 voie du TOEC, CS 27608, 31076 Toulouse Cedex 3, France; Caroline.Gibert@solagro.asso.fr; 13 Institute of Agricultural and Environmental Sciences, Estonian University of Life Sciences, Kreutzwaldi 1, 51006 Tartu, Estonia; Eve.Veromann@emu.ee

**Keywords:** crop pests, integrated pest management, natural capital, conservation biological control, landscape ecology, sentinel systems, field margins, natural enemies

## Abstract

Invertebrates perform many vital functions in agricultural production, but many taxa are in decline, including pest natural enemies. Action is needed to increase their abundance if more sustainable agricultural systems are to be achieved. Conservation biological control (CBC) is a key component of integrated pest management yet has failed to be widely adopted in mainstream agriculture. Approaches to improving conservation biological control have been largely ad hoc. Two approaches are described to improve this process, one based upon pest natural enemy ecology and resource provision while the other focusses on the ecosystem service delivery using the QuESSA (Quantification of Ecological Services for Sustainable Agriculture) project as an example. In this project, a predictive scoring system was developed to show the potential of five seminatural habitat categories to provide biological control, from which predictive maps were generated for Europe. Actual biological control was measured in a series of case studies using sentinel systems (insect or seed prey), trade-offs between ecosystem services were explored, and heatmaps of biological control were generated. The overall conclusion from the QuESSA project was that results were context specific, indicating that more targeted approaches to CBC are needed. This may include designing new habitats or modifying existing habitats to support the types of natural enemies required for specific crops or pests.

## 1. Introduction

Farming in Europe dates back approximately 5000 years and as a consequence, there has been selection pressure on animals and plants to evolve in order to survive in farmland habitats. Today, as many as 700 plant species and 2000 insect and spider species inhabit farmed areas, while many more may use them sporadically [1]. If more sustainable farming systems are going to be implemented, it is essential to maximise the contribution that some invertebrates provide towards pollinating crops, controlling crop pests, and recycling nutrients [2], while also being an important food resource for farmland birds, mammals, and other predatory arthropods. However, there is evidence that the majority of insects are in decline globally [3,4], especially in agricultural systems, attributed to the intensification of agriculture where there is widespread pesticide use [5], along with habitat loss, degradation, and fragmentation [6,7]. The steepest declines occurred in the 1970s, and some groups have since stabilised or are even starting to recover (e.g., spiders), yet some continue to decline, including pest natural enemies [5]. Action is therefore needed to encourage beneficial invertebrates in agricultural landscapes if their valuable functions are to be optimized as part of integrated pest management [8]. This may be achieved by providing all of their key resources, at the required time and in sufficient proportion, and appropriate spatial configuration to deliver benefits across fields. The key resources include: 1) shelter and appropriate microclimate for overwintering and breeding, 2) alternative prey for when pests are not present or less abundant, 3) floral resources for some taxa [9], and 4) an environment appropriate for survival with the desired vegetation structure and without harmful pest or habitat management measures. A range of seminatural habitats (SNH) already occur in agricultural landscapes and new ones are being created in agri-environment schemes, some specifically designed to provide essential resources for natural enemies [10,11], but to maximise their value we need to verify their impact [12,13]. The objective of this paper is to describe two stepwise approaches that can lead to the desired outcomes. The first is based upon gaining a detailed understanding of natural enemy ecology and resource requirements to inform habitat management or creation. The second focuses on measuring the impact of SNH on ecosystem service delivery as investigated in the QuESSA project (Quantification of Ecological Services for Sustainable Agriculture). 

**Figure 1:**
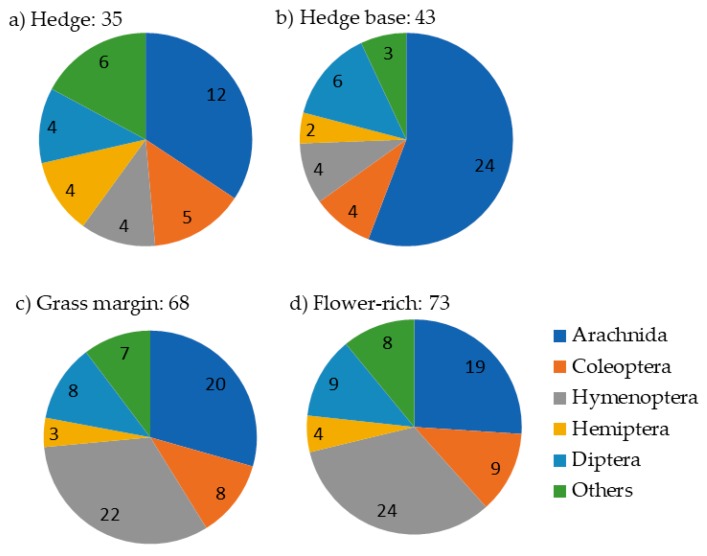
Number of natural enemy families for the predominant taxa in each habitat from a literature survey and eight databases from European projects: Buzz, Development of perennial brood rearing habitats, Farm4Bio, Farm Scale Evaluations of genetically modified herbicide tolerant crops, PEBIL (Potential for Enhancing Biodiversity on Intensive Livestock farms) (UK); Buffer zones for biodiversity of plants and arthropods: is there a compromise on width? (Denmark); The arthropods of grassy field margins (off crop) and the consequences for impact assessment of pesticides on terrestrial ecosystems (Germany); Grey partridge study (France), details in Table S1.

A wide range of seminatural habitats have already been investigated, however, the thoroughness of each investigation has been quite variable, with most focusing on evaluations of natural enemy abundance and/or species composition within the habitats [13]. Fewer studies investigated whether this led to higher numbers within the crop, while even less measured impact on pest levels or yield. Overall, the process of evaluating the value of SNH has been largely unsystematic. Nevertheless, these studies have revealed the considerable diversity that exists within such habitats, each with their own particular species composition, indicating that a multitude of habitats are likely to be more beneficial to improve natural enemy diversity and thereby to provide a more resilient biocontrol (Figure 1). Some attempts have also been made to score the habitats’ relative values based upon available information [11,12]. Unfortunately, few of the most valuable SNH are supported or are being created through agri-environment scheme funding across Europe [14], see [15,16]. Grassy strips that have some biocontrol potential [17,18] are supported in some countries’ agri-environment schemes or are compulsory as buffers for water courses (e.g., in French vulnerable zones). In some countries there has been a focus on creating flower-rich habitats for pollinators that may also be beneficial for some natural enemies, however, more specific habitats designed to support natural enemies, are not supported, with the exception of beetle banks in the UK [19] and wild flower strips in Switzerland [16,20], as far as the authors can ascertain. However, there is evidence that flower choice is extremely important in determining the suitability of any SNH for a range of natural enemies including parasitoids [21] and zoophagous hoverflies [22] and, therefore, it is necessary to create or conserve habitats with these flowers, more than a specific type of habitat.

## 2. Approaches

### 2.1. Approach Based upon Natural Enemy Ecology and Resource Requirements

**Figure 2:**
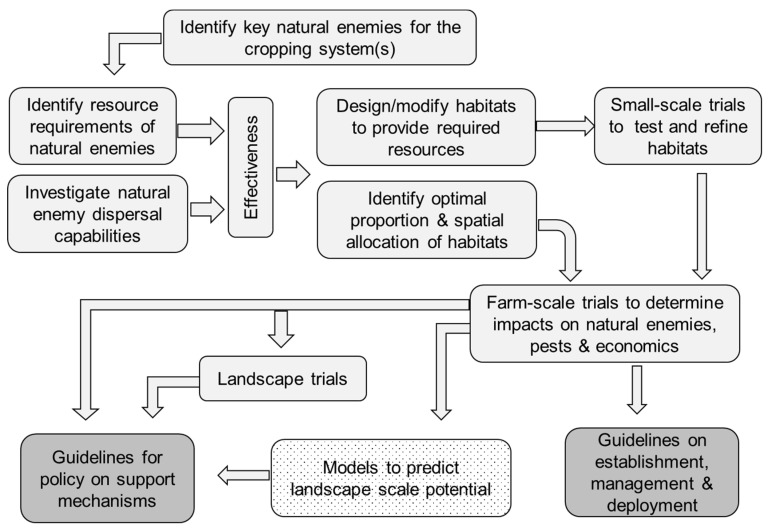
Process to exploit biocontrol potential of seminatural habitats.

The first step in the process to exploit how SNHs can increase biocontrol in agroecosystems is to identify the most important natural enemies responsible for biocontrol in the crop/system under investigation (Figure 2). Such enemies must possess as many key attributes as possible that have 1) either a high reproductive rate or are present in sufficient numbers to exhibit control at the crucial period, 2) good searching ability, not only on the host plant but across larger spatial scales, 3) host specificity or sufficient preference for the focal pest so they are not distracted by alternative prey/hosts, 4) temporal synchronization with their host, and 5) adaptation to environmental conditions within crops including management practices. The extent of these attributes will influence their effectiveness, along with a multitude of other factors, this being a complex ecosystem in which factors such as the pest’s rate of development and intra- and inter-specific competition will determine the outcome. Thus, detailed investigations of all such influences are impractical and instead experimental approaches have been developed to quantify the level of biological control and in some cases identify the types of natural enemies that provide control in cropped fields. Exclusion experiments are the most common approach [8,23] with or without camera observations, and also the level of parasitism can be measured by offering eggs or larvae that can be parasitized, e.g., [24].

For some pests, the natural enemies are already known and this information can be deduced from the literature, for example, for natural enemies of pests of cereal crops and oilseed rape, see [25,26]. Yet there remain many large gaps in our knowledge of natural enemy ecology and their effectiveness, which is not surprising given the diversity of species that can occur with crops (Figure 1). Some taxa have been relatively well studied and there is already considerable knowledge of their ecology, e.g., Carabidae [27], Coccinellidae [28], and Syrphidae [29], while for other prolific taxa little is known, e.g., predatory Diptera, excluding Syrphidae [30]. 

The next stage of the process is to determine whether there are opportunities to enhance resource requirements of these natural enemies through habitat provision and whether there are any agricultural practices that may reduce or enhance their survival, such as pesticides, tillage type, and drilling dates [5,31]. Research on resource requirements has focused on floral resources, for example, this has been investigated for parasitic wasps [21], hoverflies [32], and lacewings [33]. Engineered margins have thus been developed and tested based upon these preferences [34]. Shelter has also received some attention, with much emphasis on that required by overwintering Coleoptera [12]. The type of habitat needed by overwintering carabid beetles was investigated in the 1970s and ultimately led to the development of beetle banks, yet these have failed to gain favour because of the lack of information on their effectiveness and the perceived impracticalities of dividing larger fields. Despite these drawbacks, beetle banks are supported through agri-environment schemes in the UK [19]. Finally, whether the pests will also benefit from the habitats also requires consideration [35,36].

The value of existing SNH has also been widely investigated [12,37]. In these studies, the main focus was to measure their potential to support natural enemies rather than measuring their effectiveness to either augment natural enemy numbers within adjacent fields or across wider scales. Moreover, studies investigating the impact on pests or yields are even scarcer: for example, in a sample of 138 publications that investigated the contribution of seminatural habitats to pest control, most measured abundance of predators (70%) or parasitism (22%) within the habitats and only 22% reported pest levels, and of these only 9% measured predators and 6% parasitism [13].

The failure to measure the impact in the adjacent field or wider landscape is one of the reasons why conservation biocontrol has not been widely adopted in field crops so far. Among the possible explanations is the lack of studies distinguishing between abundance and diversity of pest natural enemy groups. It was hypothesized that diversity of natural enemies may be relevant where the pest has a complex life cycle or the pest community is patchily distributed in space and time [38]. 

Knowledge of dispersal ability and/or extent of a habitat’s influence is also valuable when determining how to deploy biocontrol habitats in the landscape. Having more mobile natural enemies allows habitats to be more widely spaced, reducing the chance that they will interfere with within-field operations performed with large machinery and reducing their susceptibility to harmful crop management operations [39]. Once the suite of the desired natural enemies and their resource requirements have been identified, then existing habitats or newly created ones may need improvement to maximise the provision of resources. This may require evaluation in field trials along with investigations of their influence on pest control if not already known. The final stage of the process is to scale up and conduct trials on-farm to ensure the habitat creation or enhancements are delivering the desired levels of pest control. For end-users this must also involve economic and risk evaluations if they are to be persuaded to adopt such an approach.

This approach relies on considerable information already being available and may be more viable if the system under investigation has relatively few pests. If several pests are important, then inevitably the list of natural enemies may become quite long and consequently may require a range of habitats or habitat types to support them, which may preclude more detailed investigations. The alternative to such detailed investigations is to focus on measuring the ecosystem service, for example, levels of pest control, a surrogate measure (e.g., predation of sentinel prey), or impact on yield without the understanding of the mechanism or agents responsible. Yield, however, is a consequence of many interacting factors and identifying the impact of biocontrol alone can be difficult. Focusing on the ecosystem service may be more appropriate when first conducting investigations of existing habitats about which relatively little is known to ascertain their potential and in comparative studies of different habitats or cropping systems. Both of these aspects were investigated in the recently completed EU FP7 QuESSA project (Quantification of Ecological Services for Sustainable Agriculture) [40]. 

### 2.2. Approach Based upon Measurement of Predation or Parasitism

**Figure 3:**
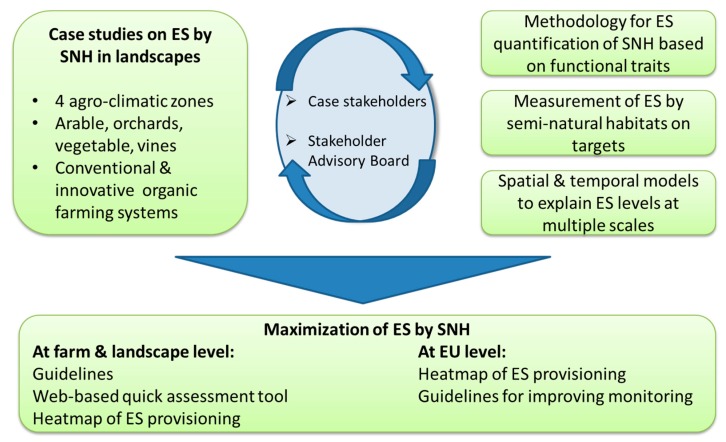
Conceptual scheme of QuESSA (Quantification of Ecological Services for Sustainable Agriculture) project approach (ES = Ecosystem Services; SNH = Seminatural Habitats).

The QuESSA project investigated the impact of seminatural habitats on a range of ecosystem services, predominantly measuring pest control and pollination, but also including services such as erosion mitigation, carbon sequestration, and aesthetic value. Research was conducted in 16 case studies across eight countries by measuring service supply in locally important crops including wheat, oilseed rape, sunflower, pumpkin, pear, olive, and vineyards. As in the previous approach, a stepwise process was followed starting with evaluations of SNH and their potential to support the ecosystem service providers, in this case natural enemies or pollinators (Figure 3). A key aim of this first stage was to identify the main habitat types that occur on farmland and develop a categorisation system that could be applied universally across Europe. The main features that were used were the shape (linear and relatively narrow or areal, e.g., block) and the predominant vegetation type (woody or herbaceous) [11]. This resulted in five categories being identified that encompassed the majority of SNH found in agricultural landscapes: 1) woody linear, 2) woody areal, 3) herbaceous linear, 4) herbaceous areal, and 5) fallow. All of these habitats were then surveyed using a standardised approach for 10 case studies located in eight countries [11]. A total of 854 species from 355 genera were found across the 539 SNH that were surveyed. The number of genera was then analysed to determine the extent of variation between the different SNH categories for the four agroclimatic zones (QuESSA Deliverable 2.4 [40]). These zones were distinct in terms of their plant composition, with the greatest differences between the maritime and Mediterranean regions. All herbaceous SNH in Europe were fairly similar in terms of genera composition, while the woody sites from the maritime regions and from the Mediterranean region have a different plant genera composition. The next stage was to develop a universal scoring system that would reflect the ecosystem service supply potential of these five habitat categories, in terms of providing pest natural enemies and pollinators. The scoring system was based on the abundance of natural enemies and pollinators captured in a selection of pan traps located within the different SNH categories. For both groups of service providers, models were developed to predict their abundances based on easy to measure vegetation traits. For pest natural enemies, vegetation-structural traits rather than flower traits were more important for predicting the value for predatory flies and parasitoids [11].

In the next main stage of the process, the actual level of ecosystem service was measured. For pest control, this entailed developing and deploying a range of sentinel prey items designed to represent either invertebrate pests or weed seeds [41]. Studies were conducted in six economically important cropping systems: oilseed rape (two case studies), pumpkin (one case study), pear (one case study), olive (one case study), winter wheat (two case studies), and vine (one case study) that varied in farming intensity. Studies were located across four European agroclimatic zones. In each of the case studies, pest control dependency on SNH was investigated in 18 study sites using standard methods. Following some initial trials to evaluate the consistency, applicability, and level of predation, a range of project-wide sentinel-preys were exposed in fields, comprising *Calliphora vomitoria* L. (Diptera: Calliphoridae) larvae exposed on the ground, *Ephestia kuehniella* Zeller (Lepidoptera: Pyralidae) eggs exposed on the ground and on the plants, *Chenopodium album* L. (Caryophyllales: Amaranthaceae), and *Poa trivialis* L. (Poales: Poaceae) seeds exposed on the ground (see QuESSA Deliverable 3.1 [40]). In addition, the predation or parasitism rate of crop specific pests was estimated at each case study site by using sentinels either of the particular pest or by measuring predation directly with predator exclusion methods. Natural enemies were recorded by using either pitfall traps for ground dwelling predators, and with pan or sticky traps for flying ones. Camera recording was used to identify predators acting on sentinel-preys in one case study. The selected sentinels were used over the following 1–2 years in each case study.

Each study site was comprised of a 1-km radius of predominantly agricultural land based around a central focal field in which the sentinels were deployed. In each country, of the 18 study sites, there were six fields that had at least one border from each of three categories of field boundary type: 1) woody, 2) herbaceous, and 3) no SNH (crop-to-crop or narrow strip of herbaceous vegetation (UK only)). Sentinels were deployed at four distances along two transects extending out into the field from the selected boundary, ensuring that none of the positions along the transect were nearer to any other boundary than the one being studied. The area within the 1-km radius was mapped using a standard protocol to determine the type, size, and location of all SNH (see QuESSA Deliverable 3.1 [40]). These data were used to investigate landscape scale impacts of SNH on sentinel predation.

For the sentinel studies there was considerable variation in the levels of predation or parasitism between sentinel types and the case studies (QuESSA Deliverables 3.3 and 5.8 [40]). In some cases, the level of predation or parasitism was more influenced by available SNH at the landscape level, while in others it was the locally bordering SNH that was more influential. Overall, no clear European trends appeared, but considerable variation between cropping systems and countries was revealed. We therefore concluded that SNH play in some situations a direct and visible role in biological control, but the complexity of the ecological mechanisms involved make it difficult to reveal possible indirect effects and thereby create general guidelines.

Besides knowing if SNH are influential in biological control, farmers and policy makers also need information on how best to deploy such features, especially new ones funded through agri-environment schemes. To optimise this process they need to know what proportion of the landscape must be devoted to such features and where they should be deployed spatially. The sentinel data were therefore further analysed to take into account the distribution of SNH within the surrounding landscape. A 2-D, landscape level, mathematical kernel approach was used [42] to weigh contributions of different seminatural habitats across a landscape and predict the level of biological control in a focal field on the basis of contributions from different habitats (e.g., SNH) in the surrounding landscape. Outcomes were used to generate pest control heat maps that indicated the level of biological control [18]. As found with the other analyses, there was considerable variability in the findings for the different sentinels and case studies. Overall though, SNH were positively associated with biocontrol services, however, the strength of the relationships was quite weak. There may be many reasons for these results, not least the sensitivity of a relatively small number of sentinels deployed within a single field to detect the contribution of SNH in the surrounding 314 ha. 

SNH also support a range of other ecosystem services and consequently there may be synergies and/or trade-offs between these services, with the potential for multi-functionality. To investigate this further, the QuESSA data from five ecosystem services (biological pest control, biodiversity conservation, carbon sequestration, landscape aesthetics, and erosion prevention) were analysed using the Landscape IMAGES framework [43] to generate, evaluate, and explore alternative landscape configurations. For the two case studies investigated, there were synergies between carbon sequestration and biological control (see QuESSA Deliverable 4.2 [40]). The scoring system outputs were also used to generate predictive European maps that indicated the potential level of biological control derived from flying natural enemies utilising SNH. These maps incorporated existing land use maps to identify the type and location of SNH which were then weighted using outcomes from the scoring system based upon the number of flying natural enemies captured in the pan traps. The score per cell was then calculated using a 2-D kernel approach as above [44]. This revealed that some highly productive agricultural regions of Europe, such as Brittany in France and the Po Valley in Italy, potentially have good levels of biological control provided by flying natural enemies, while in other highly productive regions, such as the Central Loire Valley in France and the East-Midlands in the UK, the biological control potential was estimated to be low. Finally, the quantitative data generated in QuESSA was incorporated into an existing prototype software package (the Ecological Focus Areas Calculator) [45]. This software allows the user to assess the performance of land use and landscape features on a range of ecosystem services including biological control [46].

## 3. Discussion

Despite the plethora of research into conservation biological control there are few examples of where it has been adopted in commercial practice [10]. Most of the research typically addresses one of the components described in the first approach, and although they contribute to the overall body of knowledge, outcomes from individual studies are usually not sufficient to inform or convince practitioners to implement. It is only when there is a substantial body of robust evidence, but most importantly evaluations of the economic implications and risk evaluations [47], that practical and costed recommendations for practitioners can be produced. Even then, a social network that includes the farmers, expert advisors, and agribusiness is needed if adoption is to be implemented [10], while financial reward or other incentives from the purchases of their products can also provide further incentive. Broad guidelines may not, however, be sufficient. In the QuESSA project evaluations of levels of pest control, pest control using the sentinel systems and crop specific pests revealed many varied and contrasting results (QuESSA Deliverable 3.3. [40]). The over-riding conclusion though was that the results from the case studies were very context specific, and it was not possible to provide Europe-wide guidelines on the most valuable SNH or their spatial configuration in the landscape. This is in line with other studies and five situations identified in which SNH may fail to provide pest control [39]. Thus, it would appear that there is no “quick fix” for developing conservation biocontrol habitats and that if there is a need to enhance biological control within a specific crop or cropping system, then more targeted evaluations and ultimately crop or pest orientated guidelines may be necessary. Likewise, a more systemic approach to conservation biological control, utilising a common, unifying framework was also proposed as an outcome of the EU FP7 PURE project [48]. 

Overall, the QuESSA project showed that the SNH categories are good indicators for the abundance of natural enemies, and this information may be relevant for policy makers and can help to define financial support through the common agricultural policy. Local information is crucial to effectively incorporate greening policies into ecosystem service management and to optimize the step from the presence of the pest natural enemies to the actual ecosystem service delivery to crops. This is the main aspect where future conservation biological control (CBC) research should focus. It would also be worthwhile to encourage farmers to conduct voluntary measures to protect and enhance SNH and for researchers to further investigate their role in more context-specific situations. Indeed, including farmers in the process of developing CBC and providing them with tools that they can use to monitor their level of success may help improve adoption [10]. The heatmap approach and scaling-up studies developed in QuESSA could also be used to explore how a collective, integrated (i.e., multi-disciplinary and multi-actor), and participative management for plant protection could be employed and the scale over which it is implemented. Such an approach could also be employed to aid management of wild flora and natural enemies together. 

## Supplementary Materials

The following are available online at https://www.mdpi.com/2075-4450/11/3/195/s1, Table S1: Projects from which data was extracted for compilation of Figure 1.
