# Erratum: Holland, J.M., et al. Approaches to Identify the Value of Seminatural Habitats for Conservation Biological Control. *Insects* 2020, *11*, 195

**DOI:** 10.3390/insects11050272

**Published:** 2020-04-29

**Authors:** John M. Holland, Philippe Jeanneret, Anna-Camilla Moonen, Wopke van der Werf, Walter A. H. Rossing, Daniele Antichi, Martin H. Entling, Brice Giffard, Herman Helsen, Mark Szalai, Carlo Rega, Caroline Gibert, Eve Veromann

**Affiliations:** 1 Farmland Ecology Unit, Game and Wildlife Conservation Trust, Fordingbridge SP6 1EF, UK; 2 Agroecology and Environment, Agroscope, CH-8046 Zurich, Switzerland; philippe.jeanneret@agroscope.admin.ch; 3 Scuola Superiore Sant’Anna, Agroecology Group, Institute of Life Sciences, Via Santa Cecilia 3, 56127 Pisa, Italy; moonen@sssup.it; 4 Wageningen University & Research, Crop Systems Analysis, Droevendaalsesteeg 1, 6708PB Wageningen, The Netherlands; wopke.vanderwerf@wur.nl; 5 Wageningen University & Research, Farming Systems Ecology, Droevendaalsesteeg 1, 6708PB Wageningen, The Netherlands; walter.rossing@wur.nl; 6 Centre for Agri-environmental Research “Enrico Avanzi”, University of Pisa, Via Vecchia di Marina 6, 56122 San Piero a Grado, Pisa, Italy; daniele.antichi@unipi.it; 7 iES Landau, Institute for Environmental Sciences, University of Koblenz-Landau, Fortstr. 7, D-76829 Landau, Germany; entling@uni-landau.de; 8 Bordeaux Sciences Agro, INRAE, UMR 1065 Santé et Agroécologie du Vignoble, University of Bordeaux, F-33170 Bordeaux, France; brice.giffard@gmail.com; 9 Wageningen University & Research, Wageningen Plant Research, Lingewal 1, 6668LA Randwijk, The Netherlands; Herman.Helsen@wur.nl; 10 Plant Protection Institute, Szent Istvan University, Pater K. str. 1, Szent Istvan University, H-2100 Gödöllő, Hungary; Szalai.Mark@mkk.szie.hu; 11 European Commission, Joint Research Centre (JRC), Via E. Fermi 2749, 21027 Ispra, VA, Italy; carlo.rega@ec.europa.eu; 12 SOLAGRO, 75 voie du TOEC, CS 27608, CEDEX 3, 31076 Toulouse, France; Caroline.Gibert@solagro.asso.fr; 13 Institute of Agricultural and Environmental Sciences, Estonian University of Life Sciences, Kreutzwaldi 1, 51006 Tartu, Estonia; Eve.Veromann@emu.ee

We would like to make the following correction to the published paper [1] as affiliations 9, 10 and 11 incorrect. They should be as follows:

^9^  Wageningen University & Research, Wageningen Plant Research, Lingewal 1, 6668LA Randwijk, The Netherlands; Herman.Helsen@wur.nl
^10^  Plant Protection Institute, Szent Istvan University, Pater K. str. 1, Szent Istvan University, H-2100 Gödöllő, Hungary; Szalai.Mark@mkk.szie.hu
^11^  European Commission, Joint Research Centre (JRC), Via E. Fermi 2749, 21027 Ispra, VA, Italy; carlo.rega@ec.europa.eu

The change does not affect the scientific results. We apologize for any inconvenience brought to the readers.
