# Supplementary material for: Approaches to Identify the Value of Seminatural Habitats for Conservation Biological Control
Source: Insects. 2020 Mar 20;11(3):195. doi: 10.3390/insects11030195 (PMC7143897; doi:10.3390/insects11030195)
Supplement: Supplementary file 1 [file insects-11-00195-s001.pdf]

Table S1. Projects from which data was extracted for compilation of Figure 1.

Comparison of new and existing agri-environment scheme options for biodiversity enhancement on arable land - Buzz project (UK).<br>Six arable farms, one field per farm, compared five field margin treatments replicated along two margins and mid-field wild bird seed mixture. Transect counts of bees and butterflies made between May and September, suction (using Vortis) and sweep net samples collected in July, pitfall trapping April/May and October each year from 2002-06.
Development of perennial brood rearing habitats project - Game & Wildlife Conservation Trust (UK).<br>Comparison of three wildflower seed mixes in a fully replicated trial established in two separate fields in Dorset. Treatments were sampled using a Dvac suction sampler in late June in 2009 and 2010.
Managing uncropped land in order to enhance biodiversity benefits of the arable farmed landscape - Farm4bio project (UK).<br>This project included 28 farms with a range of target habitats, but the number of habitats and samples taken per year varied between farms. Suction sampling (using Vortis) was conducted mid-summer and transect counts of pollinators were made in June and August annually for 3 years (2008-10).
Farm Scale Evaluations of genetically modified herbicide tolerant crops - FSE (UK).<br>Only data collected in the verge (habitat adjacent to the hedge base) for the 65 Winter Oilseed rape control fields was included as these fields were distributed nationally, unlike some of the other crops that were more regional. Transect counts of pollinators were made in April, May, June and July, suction samples (using Vortis) in June and August, and pitfall trapping in April/May, June/July and September/October. 21 fields were sampled in 2000, 29 in 2001 and 15 in 2002.
Buffer zones for biodiversity of plants and arthropods: is there a compromise on width? (Denmark).<br>One farm, four fields, two margins per field with a range of treatments assessed in May, June and July during 2008. Assessments including in review were those conducted in the hedge using beating trays and in the base for bees and butterflies using transect counts, and for foliage and ground inhabiting invertebrates using pitfall traps and sweep netting.
The arthropods of grassy field margins (off crop) and the consequences for impact assessment of pesticides on terrestrial ecosystems (Germany).<br>Three regions, 24 sites per region of typically tall ruderal oat grass. Each site was assessed using soil cores for Collembola, pitfall traps from April to October, visual observations of Coccinelidae, Orthroptera and pollinating insects from May to October.
Game & Wildlife Conservation Trust - Grey partridge study (France).<br>Invertebrates were collected annually (2001–2004) in field margin habitats on two farms in Normandy, using a Dvac suction sampler.
